# Effects of Pretreatment with Ionic Liquids on Cellulose Hydrolysis under Hydrothermal Conditions

**DOI:** 10.3390/molecules24193572

**Published:** 2019-10-03

**Authors:** Toshitaka Funazukuri, Shingo Ozawa

**Affiliations:** Department of Applied Chemistry, Chuo University, 1-13-27 Kasuga, Bunkyo-ku, Tokyo 112-8551, Japan; bagcar98@yahoo.co.jp

**Keywords:** cellulose, ionic liquid, hydrothermal, imidazolium, filter paper, hydrolysis, rate

## Abstract

Hydrothermal hydrolysis in hot pressurized liquid water (HPLW) is attractive for biomass conversion into valuable products because it achieves high reaction rates without catalysts and additives. The hydrothermal hydrolysis of high crystalline cellulose requires higher reaction temperature than polysaccharides having low crystallinity. It can be expected to increase the reaction rate or decrease temperature by decreasing the crystallinity. In the present study ashless filter paper as a fibrous pure cellulose sample was pretreated with ionic liquids (ILs) such as imidazolium chloride ILs containing alkyl side chains ranging from two to six carbons, and with an aqueous solution of bis(ethylenediamine ammonium) copper (BEDC). Herein, the pretreatment with ILs was to regenerate filter paper: dissolving in ILs at 373 K for 120 min or in an aqueous BEDC solution at room temperature, precipitating by adding water, washing the solid, and then drying. Subsequently, the pretreated filter paper samples were hydrolyzed at 533 K and 5.0 MPa in HPLW in a small semi-batch reactor, and the effects of the pretreatment with ILs or BEDC on reaction rates and product yields were examined. While the crystallinity indexes with all ILs and BEDC after the pretreatments decreased to 44 to 47 from the original sample of 87, the reaction rates and product yields were significantly affected by the IL species. At 533 K and 5.0 MPa, the dissolution rate with [AMIM][Cl] was nine times as fast as that for untreated sample.

## 1. Introduction

Cellulose is the most terrestrial abundant biomass on Earth, with the potential for conversion into products such as chemical feedstock, feed, dietary supplements, and fuel. In particular, glucose converted from cellulose leads to bioethanol by fermentation as the production from non-food materials [1]. Thus, many degradation processes that convert cellulosic materials into useful products such as mono-saccharides as well as cello-oligosaccharides have been developed [1,2,3,4,5,6,7,8,9,10]. Glucose and cello-oligosaccharides are important for biofuel, and pharmaceutical and nutraceutical industries, respectively.

Cellulose can be hydrolyzed by an enzyme or acid to produce glucose and cello-oligosaccharides. Many studies on both of these hydrolysis methods have been reported. Enzymatic hydrolysis results in highly selective formation of useful products, but involves slow reaction rates. Acid hydrolysis results in less selective production, but involves relatively high reaction rates. In addition to both of these conventional methods, hydrothermal hydrolysis (hydrolytic decomposition in pressurized liquid hot water) can convert cellulose or other polysaccharides into glucose and oligosaccharides without the need for additives, or with an acid at very low concentrations (i.e., < 1 wt.%) [9,10] with relatively high selectivity and high reaction rates.

Hydrothermal hydrolysis of cellulose is commonly conducted at temperatures above 200 °C. Since the products such as monosaccharides and oligosaccharides are more readily decomposed than the original cellulose under hydrothermal conditions, a flow reactor needs to be used so that the products are removed from the reactor once the products are formed [11].

Microcrystalline cellulose powder is frequently used for studies on hydrothermal hydrolysis of cellulosic samples because of its amenability for continuous feeding [9]. However, cellulosic biomass resources, such as agricultural waste, wood, grasses, medicinal plants, and waste newspaper, are fibrous. For practical purposes, fibrous cellulosic samples need to be examined for cellulose conversion. Therefore, filter paper was used as a sample of fibrous pure cellulose in the present study, to eliminate the effects of other components such as hemicellulose and lignin.

In contrast to starch, which contains α-1,4-glucosidic linkages, cellulose consists of β-1,4-glucosidic linkages, and contains intra- and intermolecular hydrogen bonds, which makes it highly crystalline. Previous studies on the hydrolysis of starch under hydrothermal conditions [12,13,14] indicated that starch was solubilized and hydrolyzed at much lower temperatures than those needed for cellulose such as microcrystalline cellulose powder, filter paper, and cotton cellulose [9,10]. Therefore, more severe degradation conditions, such as higher reaction temperatures and the presence of an acid, are required [9,10,11]. Reduction in the crystallinity of cellulose allows it to be converted more efficiently into useful products under milder reaction conditions while suppressing the formation of unfavorable secondary products.

Since the discovery by Swaltloski et al. that cellulose can be dissolved in ionic liquids (ILs) [15], various studies on the dissolution of cellulosic materials and the hydrolysis of cellulose dissolved in ILs have been conducted [16,17]. Moreover, Li et al. (2008) [18] studied the hydrolysis of cellulose using a combination of a mineral acid and an IL(1-Butyl-3-methylimidazolium chloride [BMIM][Cl]), and obtained a total reducing sugar yield of 77% at 100 °C in 540 min. Rinaldi et al. (2008) [19] studied the depolymerization of cellulose in [BMIM][Cl] with ion-exchange resin, Amberlyst 15 DRY as a catalyst, and obtained a reducing sugar yield of 28% at 100 °C in 5 h. In the presence of acidic solid catalysis in ionic liquid hydrolytic conversions of cellulose to glucose and further decomposition products were also studied [20,21]. Although these methods are improvements over the use of mineral acid or acid catalyst alone, they are limited by the nature of the catalyst and its activity under the given conditions.

In addition to the use of mineral acids or solid acid catalysts, a number of studies on enzymatic hydrolysis of cellulose pretreated with ILs have been reported [22,23,24,25,26,27,28,29]. The IL pretreatment clearly enhanced yields of glucose and oligosaccharides. However, the inhibitor effects of ILs on enzymatic activity lead to drawbacks. Thus, studies on the production of enzymes with wide tolerance limits to ILs are currently being performed [30,31]. Moreover, since ILs have extremely low vapor pressures, the separation of hydrolytic products from the resultant solutions containing ILs, and the recovery and purification of ILs and/or products may not be easy. Thus, it is better to remove the IL used for the pretreatment prior to the subsequent hydrolysis processes.

Recently, we carried out the hydrolysis of cellulose, e.g., cotton cellulose, in a dilute aqueous formic acid solution in a semi-batch reactor, which yielded 36.6% of glucose on carbon weight basis, and sugars including glucose, fructose and cello-oligosaccharides with degree of polymerization (DP) = 2 to 9 of 83.8% under hydrothermal conditions [9]. Hirajima et al. found the use of various aqueous dilute organic acid solutions accelerated the rates of hydrolysis for pure cellulose filter paper under hydrothermal conditions [10]. However, amorphous cellulose formed by treatment with ILs is expected to be effectively depolymerized, even in the absence of acids. Thus, the objective of this study is to investigate the effects of ILs as a pretreating reagent on reaction rates and product distributions from hydrolysis in water under hydrothermal conditions without additives in a small semi-batch reactor.

## 2. Results and Discussion

### 2.1. Dissolution Rate in Hydrothermal Hydrolysis

**Figure 1:**
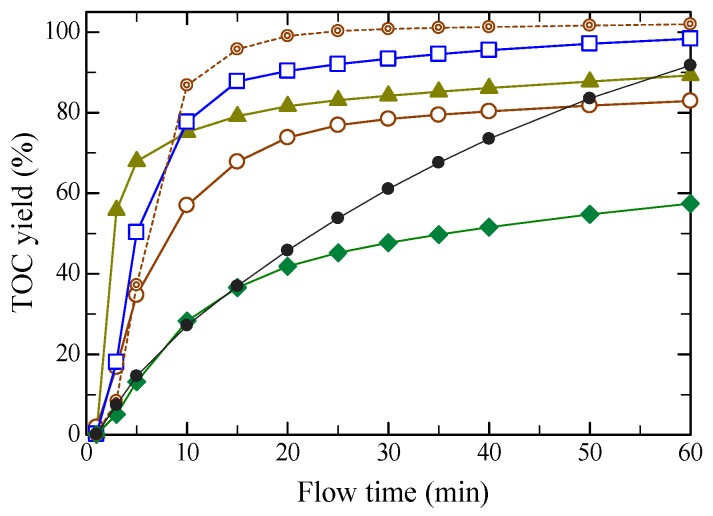
Comparison of the effects of ionic liquids (ILs) on time change in TOC yield from hydrothermal hydrolysis of regenerated solid filter paper, which was obtained as follows: dissolution with IL at 373 K for 120 min or bis(ethylenediamine ammonium) copper (BEDC) at room temperature for 30 min, precipitating by adding water, washing and then drying. Hydrolysis condition was at 533 K and 5.0 MPa by flowing water at 15.0 mL/min. ILs used for dissolving filter paper are: 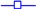: [EMIM][Cl], 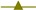: [AMIM][Cl], 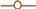: [BMIM][Cl], 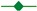: [HMIM][Cl], 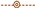: BEDC, 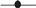: untreated.

Figure 1 shows TOC (total organic carbon) yields vs. flow time from the hydrothermal hydrolysis of regenerated filter paper, together with that without the pretreatment. Herein, regenerated filter paper sample was solid cellulose obtained from the dissolution with ILs at 373 K for 120 min or with bis(ethylenediamine ammonium) copper (BEDC) at room temperature for 30 min, precipitating by adding water, washing the solid with water and then drying. Note that TOC yield at a certain flow time was obtained by cumulating the TOC value in each fraction from the start to the flow time. The TOC yield from dissolved with [EMIM][Cl] at 60 min was initially the highest among all of the ILs studied, and decreased with increasing number of carbon atoms in the alkyl side chain. Note that the side chain of [AMIM][Cl] has a double bond, and those of the other ILs are saturated. While the effect of the double bond is not clarified, the reaction rate was the fastest, and the final TOC yields were between [EMIM][Cl] and [BMIM][Cl]. Note that the abbreviations of ILs, the chemical structures and purities are described later (Figure 1 and Table 2, respectively).

In our previous study [11] the reproducibility for TOC and product yields was examined by carrying out the reaction three times at the identical reaction conditions. Using the same reactor that was used in the study [11], at every 3 to 15 min the authors measured the yields of TOC, glucose, fructose and cello-oligosaccharides DP = 2 to 9, and further decomposition products such as levoglucosan and 5-hydroxymethyl furfural in each fraction of the effluent from the reactor, heated at 523 K and 10 MPa by flowing water at 15 mL/min. As a result, the time changes in TOC and glucose were quite reproducible within ±3% over a nearly entire conversion range, and the reproducibility of total sugar yields from glucose to cello-oligosaccharides up to DP = 9 were within ±5% [11].

While the TOC yield with [AMIM][Cl] at 60 min was lower than that with [EMIM][Cl], the increase in rate of TOC yield with [AMIM][Cl] was faster in the initial stage. Note that [AMIM][Cl] has a double bond and the other ILs do not. The pretreatment by ILs, except for [HMIM][Cl], accelerated dissolution rates (in terms of TOC) and the rates depended on IL species, as compared to that without the pretreatment. For [HMIM][Cl], almost no effects were observed from the initial stage to 20 min, and lower TOC yields were generated thereafter. The TOC yields at 60 min, where the TOC values nearly leveled off, increased in the order of [HMIM][Cl] (6) < [BMIM][Cl] (4) < [AMIM][Cl] (3) < [EMIM][Cl] (2), where the numbers in parentheses designate the number of carbon atoms in the alkyl side chain. It is interesting that shorter alkyl side chains resulted in higher final TOC yields or conversion. While the reason for this is not clear, one possibility is that the cellulose molecules may be arranged such that they could be more favored to contact water molecules due to less hydrophobicity of the shorter alkyl side chains. Another possibility is that the hydrogen bonds were interrupted to a greater extent by ILs with shorter alkyl side chains. Although the dissolution rate with [AMIM][Cl] was the fastest in the ILs studied, the final TOC value was lower than those with [EMIM][Cl] and without pretreatment. The TOC yield for filter paper pretreated with BEDC is also interesting. An aqueous BEDC solution is well known to dissolve cellulose, and it is commonly used as a dissolution reagent to measure the molecular weight distribution of cellulose via the ultimate viscosity [32]. Although the dissolution rate with BEDC was similar to that of [EMIM][Cl], the TOC yields were higher.

**Figure 2:**
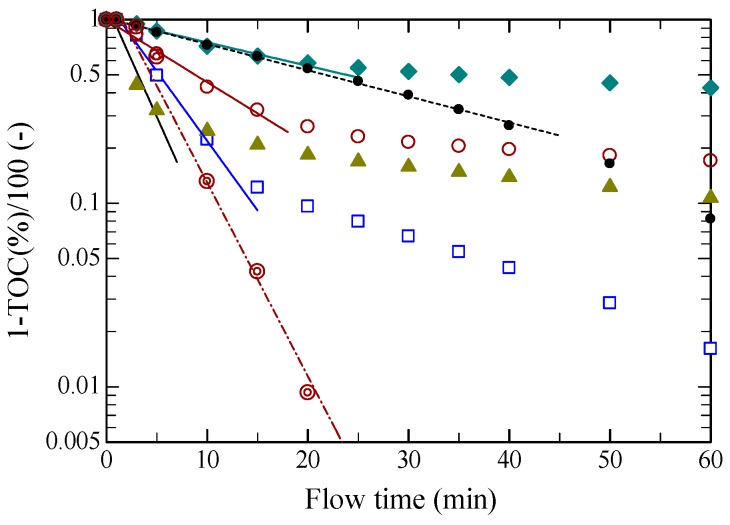
Dissolution rates in terms of TOC yield for regenerated solid filter paper: dissolved with various ILs or BEDC, precipitated, washed and then dried, together with original filter paper, all hydrolyzed in water at 533 K and 5.0 MPa for flow time up to 60 min in a semi-batch reactor. The key is the same as that in Figure 1.

Figure 2 shows [1 − TOC(%)/100] vs. flow time, hydrolyzed at 533 K and 5.0 MPa, as semi-logarithmic plots for pretreated samples with various ILs, together with those with BEDC and without pretreatment. The rate can be represented by the first order reaction kinetics, followed by a first order reaction with lower reaction rates, as

(1) $$\frac{dy}{dt}=-ky$$

where *y* is the unreacted portion of the sample defined as $y=1-\frac{\mathrm{TOC}\left(\%\right)}{100}$ and *k* is the first order rate constant (1/min) in the initial stage. In all cases in the initial stage, the dissolution rate, i.e., decreasing rate of unreacted mass, was expressed with first order reaction kinetics. The rate constants (1/min) at 533 K and 5.0 MPa were 0.032, 0.029, 0.078, 0.174, 0.283 and 0.243 for untreated, [HMIM][Cl], [BMIM][Cl], [EMIM][Cl], [AMIM][Cl], BEDC, respectively. The rate with [AMIM][Cl] was almost 9 times as fast as that for untreated sample. The rates in the initial stage increased in the order of [HMIM][Cl] < [BMIM][Cl] < [EMIM][Cl] < [AMIM][Cl], although the crystallinity indexes for regenerated samples pretreated with all ILs were almost the same, as described later. The case of [HMIM][Cl] was almost equal to the untreated case, and the reaction was retarded for longer flow times, whereas the dissolution proceeded at a constant rate for the untreated sample. Thus, [HMIM][Cl] was not effective in dissolution rates because the OH groups in cellulose polymers could be not well miscible with ILs containing longer alkyl side chains. The rate with [BMIM][Cl] was faster than that with [HMIM][Cl], and Equation (1) holds at higher conversions than that with [HMIM][Cl]. The rate with [EMIM][Cl] was faster than that with [BMIM][Cl], but lower than that with [AMIM][Cl]. The initial rate with [AMIM][Cl] was faster than that with [EMIM][Cl], but the second stage of dissolution was lower. This may result from the double bonds in [AMIM][Cl], while shorter alkyl side chains are more effective for saturated ones. It is interesting that the rate with BEDC was the highest, almost equal to that with [AMIM][Cl] with a longer induction time. The dissolution rate was expressed by Equation (1) over nearly the entire conversion range. In the second stages, the slopes for all of the ILs were less steep and the slopes for [HMIM][Cl], [BMIM][Cl], and [AMIM][Cl] were similar except for [EMIM][Cl]. Although the reaction mechanisms are not known, less reactive portions may react in the second stage.

Solubilities of cello-oligosaccharides, especially those with higher DPs in water under hydrothermal conditions, were much lower than those of malto-oligosaccharides obtained from the hydrolysis of starch, and the dissolution reaction should include degradation of cello-oligosaccharides. The breakage of hydrogen bonds by ILs may be significantly affected by the IL species themselves, namely depending on their compatibility with cellulose molecules.

### 2.2. Scanning Electron Microscope

**Figure 3:**
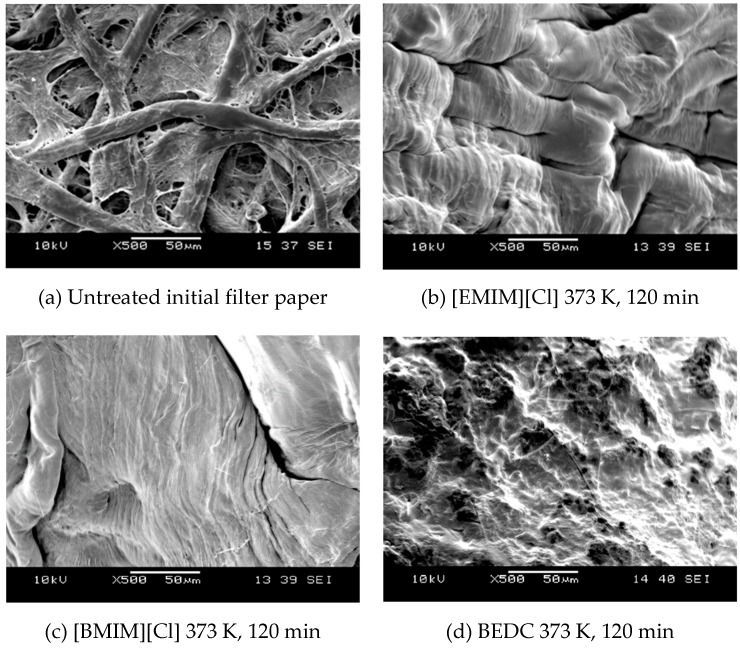
SEM images of filter paper sample, (**a**) untreated, treated with (**b**) [EMIM][Cl], (**c**) [BMIM][Cl] and (**d**) BEDC.

SEM images of (a) original filter paper, (b) that treated by [EMIM][Cl], (c) [BMIM][Cl] and (d) BEDC at 373 K for 120 min are shown in Figure 3. After the treatment with [BMIM][Cl], cellulose fiber was dissolved followed by precipitation in water, and the fibers seemed to be fused for all ILs and BEDC. However, the morphology of the surfaces was different.

### 2.3. Product Yields

**Figure 4:**
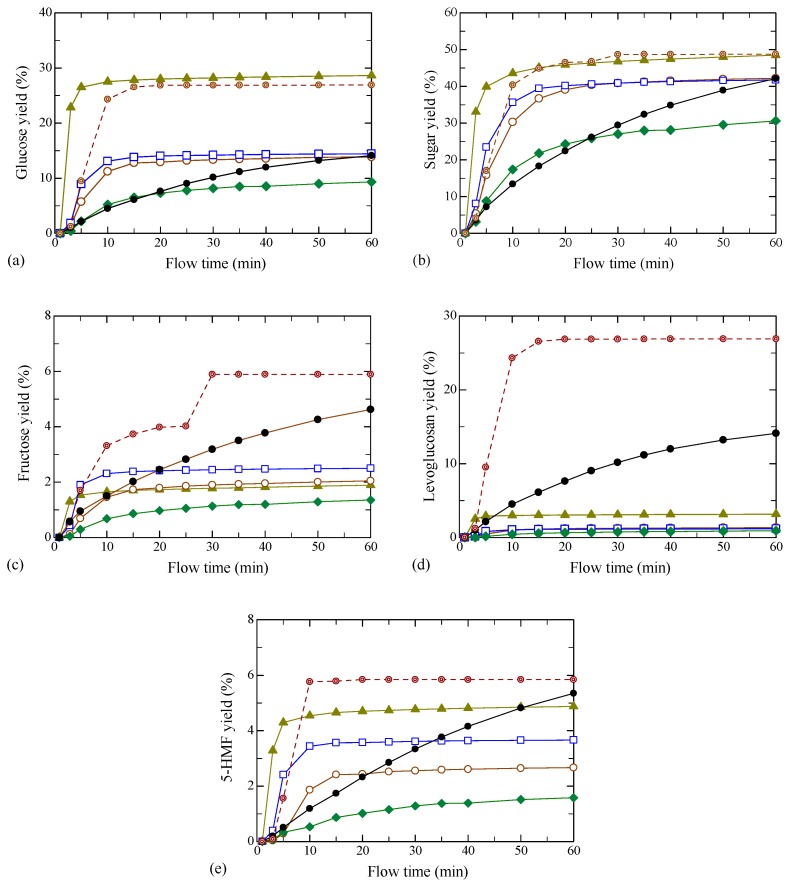
Yield over flow time for filter paper pretreated with ILs at 373 K for 120 min or with BEDC at room temperature for 120 min, followed by hydrolysis at 533 K and 5.0 MPa in water, together with that without pretreatment by hydrolysis at 533 K and 5.0 MPa in water. (**a**) glucose, (**b**) sugar, (**c**) fructose, (**d**) levoglucosan and (**e**) 5-HMF. 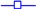: [EMIM][Cl], 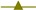: [AMIM][Cl], 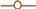: [BMIM][Cl], 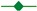: [HMIM][Cl], 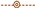: BEDC, 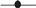: untreated.

Figure 4a depicts the glucose yield vs. flow time under the same hydrolysis conditions as in Figure 1. Note that the yield at a certain flow time was obtained by summing up the yield in each fraction from the start to the flow time, the same as TOC yield. The dissolution rate of glucose with [AMIM][Cl] was the fastest as seen for TOC, and the yield was also the highest. The yields for the various ILs showed a similar tendency to those observed in Figure 4a, although they were lower. The yields with BEDC were slightly slower to reach the plateau value, but the value was almost the same as that with [AMIM][Cl]. The yield for untreated sample was slower than for those with all of the ILs excluding [HMIM][Cl]. The ILs with higher glucose yields showed faster reaction rates and reached faster plateau values. In contrast to those treated with ILs and BEDC, the yield without pretreatment gradually increased with time. Figure 4b shows the yields of sugar, which were obtained by the total yields of glucose and cello-oligosaccharides with DP = 2 to 9 and fructose. The tendency in the plots of sugar yield vs. flow time was similar to that for glucose. For fructose shown in Figure 4c, the yields were also similar to those for glucose, but the yield for BEDC was much higher than those in other cases. Figure 4d,e shows the yields of further decomposition products, levoglucosan and 5-HMF. 5-HMF may be produced via levoglucosan [33]. The 5-HMF yields were higher than those without pretreatment in the initial stage—up to 15 min, when the yields plateaued—except for [HMIM][Cl]. In contrast, the yields of levoglucosan were lower than those without the pretreatment. It can be expected that further decomposition reaction from levoglucosan to 5-HMF may proceed when the pretreatments with ILs and BEDC were performed, while the 5-HMF yield gradually increased without pretreatment.

Table 1 shows the yields of TOC and major products with various ILs and BEDC pretreatments at 533 K and 5.0 MPa for 60 min, together with those without pretreatment. When the reaction was completed, no residual solid was found in any case, except for [HMIM][Cl]. The TOC yields at 60 min reached higher than 80%, and cellulose was almost perfectly converted to soluble components. The yields of unidentified products were obtained by subtracting the total yields of products measured from the TOC yield. The value with [EMIM][Cl] was high (49.3%), and the others ranged from 30 to 40%, except for [HMIM][Cl] and BEDC. According to the mass spectra measured by MALDI-TOF-MS of cello-oligosaccharides with DP up to 30 or higher, those with DPs higher than 9 were expected to be produced. Note that levulinic acid, sometimes reported as a further decomposition product from monosaccharides [33], was not detected in the present study. The authors found that the yields of glucose and cello-oligosaccharides with DP = 2 to 9 were proportional to TOC yield, and the slopes in plots of product yield vs. TOC yield were not affected by hydrolysis temperature [9,10,11].

**Figure 5:**
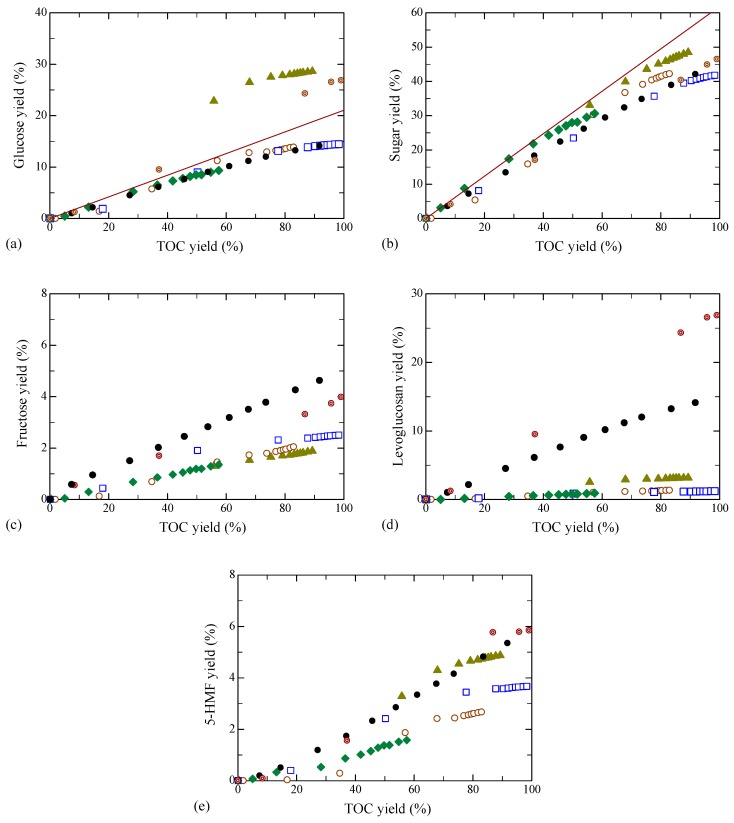
Relationship between product and TOC yields. (**a**) Glucose, (**b**) sugar, (**c**) fructose, (**d**) levoglucosan, and (**e**) 5-HMF yield. The data and key are the same as in Figure 1.

Figure 5 examines the relationship between the product yield and TOC yield for various ILs pretreatments. In Figure 5a,b, the yields of (a) glucose and (b) sugars were almost proportional to TOC yield over nearly the entire range of conversion. Moreover, the plots for glucose with [EMIM][Cl], [BMIM][Cl] and [HMIM][Cl] as well as that without pretreatment could be expressed by a single line, except for [AMIM][Cl]. For BEDC the yields up to TOC yield of 40% were also consistent. While the sugar yields scattered somewhat, the plots agreed in all cases, even with [AMIM][Cl] and BEDC. Fructose is known to be produced via isomerization of glucose [34]. The plots with ILs showed similar tendencies to those for glucose, as can be seen in Figure 5c. The differences were that the yields without the pretreatment were higher and those with [AMIM][Cl] were almost the same as those with the other ILs. Levoglucosan and 5-HMF are the further decomposition products from monosaccharides, glucose and fructose, and are produced by subtracting one and three water molecule(s) from the monosaccharides, respectively [34]. Thus, the yields were significantly affected by the reaction conditions. The yields of levoglucosan with all of the ILs were much lower than those without pretreatment and BEDC, as can be seen in Figure 5d. The slopes in plots of yields of 5-HMF vs. TOC yield were categorized into two groups: high and low. In Figure 5e, the former contained [AMIM][Cl], untreated, and BEDC, and the latter contained [EMIM][Cl], [BMIM][Cl] and [HMIM][Cl]. Further studies on the reaction mechanisms are required to clarify the reason.

**Figure 6:**
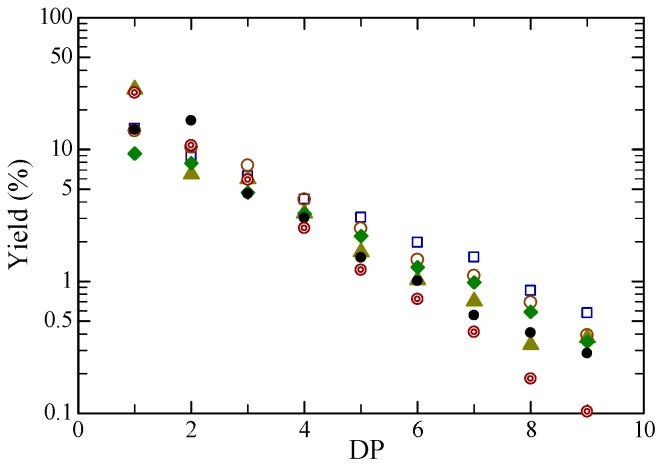
Product yield vs. degree of polymerization (DP) for pretreatment with various ionic liquids and BEDC, and untreated. The key is the same as in Figure 1.

Figure 6 shows product yield vs. DP with various ILs and BEDC, together with untreated cellulose. The yields decreased with increasing DP for all samples as seen for filter paper with water [9,10,11], whereas the slopes were slightly different. It is interesting that the product yields for untreated cellulose were almost the same as those for various ILs and BEDC.

### 2.4. Effect of Pretreatment Time for [BMIM][Cl]

**Figure 7:**
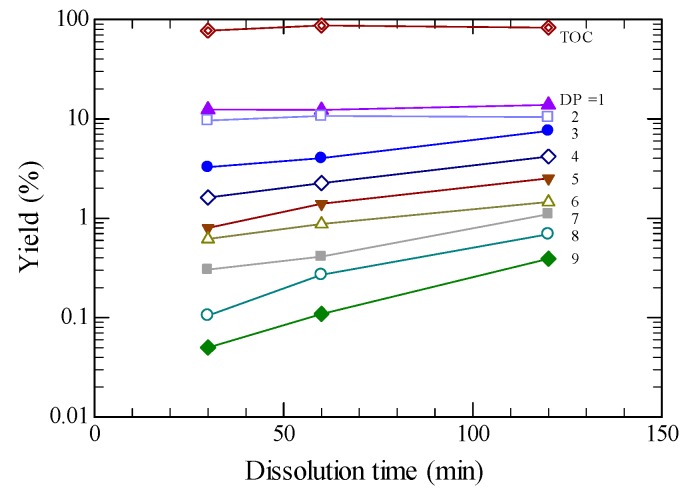
Effects of dissolution time on product yields and TOC.

In the present study the dissolution time with ILs was mainly fixed to be 120 min, sufficiently long. However, the effects of dissolution time on product yields were tested. Figure 7 shows effects of dissolution time on product yields for [BMIM][Cl] at 373 K, hydrolyzing at 533 K and 5.0 MPa. While the yields of TOC, glucose and cellobiose were almost constant with increasing dissolution time, the increases in the yields for higher DPs were more evident. Since more amorphous cellulose could be produced with increasing dissolution time, the production of higher molecular weight products may be affected more.

## 3. Materials and Methods

### 3.1. Chemicals

**Figure 8:**
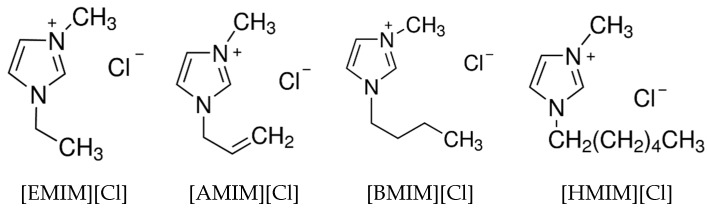
Chemical structures of ILs.

The ILs used are imidazolium chlorides containing alkyl side chains containing 2 to 6 carbons. Their chemical structures are presented in Figure 8. The cellulose used in the present study is ashless filter paper (No. 7, Advantec, Tokyo, Japan). The chemicals employed were obtained from Sigma-Aldrich, and those with their purities are listed in Table 2. All of the chemicals were used without further purification.

### 3.2. Experimental Procedures

#### 3.2.1. Pretreatment with Ionic Liquids and BEDC

Dried cellulose (0.5 g) and IL (4.5 g) were loaded in a Teflon^®^ test tube (inner diameter: 10 mm and length: 10 mm, ca. 8 mL) which was then capped. The tube was immersed in an oil bath, whose temperature was maintained at 373 ± 1 K. After a prescribed time elapsed, the tube was removed from the oil bath, and then cooled by water. The tube was opened and ultra-pure water (made by Millipore) was poured in the tube. The products and solution were recovered in a beaker and washed with ultra-pure water in the reactor. The product solutions were well stirred, and then centrifuged at 100 rpm for 10 min. The supernatant was removed by suction filtration. Again, 30 mL ultra-pure water was added to the beaker, and the mixture was stirred well, centrifuged, and the supernatant was removed by suction filtration. This process was conducted in its entirety three times. The residual solid, regenerated IL-treated filter paper, was separated by a glass filter, and dried at 333 K for 72 h. Then, the residual solid was hydrolyzed in the semi-batch reactor under hydrothermal conditions.

For the BEDC solution, dried cellulose (0.5 g) and 0.5 M aqueous BEDC solution (100 mL) were loaded in a flask and stirred at room temperature for 30 min. To precipitate the cellulose, a 1 M sulfuric acid solution (110 mL) was dropped into the flask containing cellulose and BEDC. The precipitated cellulose was washed well with ultrapure water and the water was removed by vacuum filtration. The cellulose solid thus obtained was washed again with ultra-pure water, and then filtered. This process was repeated four times, and then dried at 333 K for 72 h. Then, the cellulose solid so obtained was further hydrolyzed in the semi-batch reactor under hydrothermal conditions.

The nitrogen content in the residual solid (mainly cellulose) was analyzed by a CHN coder (model MT-5, Yanaco, Kyoto, Japan) to examine the IL remaining in the treated filter paper. The water content was estimated by thermogravimetric analysis.

#### 3.2.2. Hydrolysis in a Semi-Batch Reactor

The IL-treated filter paper was hydrolyzed in a semi-batch reactor. The reactor set-up and procedure have been described elsewhere [10,11]. Briefly, about 0.5 g of dried treated cellulose sample (water content of 1–2 wt.%) was softly wrapped with a stainless-steel screen (200 mesh) in a small tubular reactor (3.6 mL) made of stainless steel (SUS 304, inner diameter of 6.7 mm and length of 8 mm). Distilled water was added to the entire line, from the preheating column, reactor, heat exchanger made of coaxial tubes to back-pressure regulator. At time zero, the reactor at room temperature was immersed in a molten-salt bath whose temperature was maintained within ±2 K of the prescribed temperature, and distilled water was fed into the reactor by HPLC pumps (JASCO corporation, Tokyo, Japan) at 5 MPa and a flow rate of 15 mL/min measured at room temperature. Over a certain time period (2 to 15 min, longer intervals as reaction times increased), the solution was recovered from the effluent from the back-pressure regulator. All of the effluents were recovered by combining all of the fractions. In the previous study, the temperature of the fluid in the reactor was found to reach the prescribed temperature within 30 s, as determined using a thermocouple placed in the reactor [11]. The fluid residence times in the reactor were 20 to 30 s, directly measured by a trace response technique [11].

#### 3.2.3. Analyses

The organic carbon content in the product solution was measured by a TC analyzer (Model: TC 5000A, Shimadzu, Kyoto, Japan). The mono- and oligosaccharides were identified by TOF-MS (Model AXIMA-CFR, Shimadzu). The yield of glucose, cello-oligosaccharides with DP = 2 to 9, fructose, 5-methyl-2-furaldehyde, and anhydro-β-glucose were measured by an HPAEC (LC30, Dionex, Dionex Japan, Tokyo) with a CarboPac PA1 column, using 0.05 M aqueous sodium hydroxide solution and a mixture of 0.5 M sodium acetate and 0.05 M sodium hydroxide solution as mobile phases. Crystallinity indices of celluloses untreated and treated with ILs were measured using an X-ray diffractometer (model Rint 2000, Kigaku, Tokyo, Japan).

##### Degree of crystallinity

The degrees of crystallinity *X*_CR_ were determined using Equation (2) by the method of Segal et al. [35]:

(2) $$X_{\mathrm{CR}}\left[\%\right]=\frac{I_{\mathrm{CR}}-I_{\mathrm{AM}}}{I_{\mathrm{CR}}}\times 100$$

where *I*_CR_ and *I*_AM_ are the X-ray diffraction intensities of cellulose type I and amorphous cellulose, respectively.

##### Definition of yield

The yields of product and total organic carbon were defined by Equations (3) and (4), respectively.

(3) $$\mathrm{product}\;\mathrm{yield}\;\left(\%\right)=100\times \frac{\mathrm{carbon}\;\mathrm{in}\;\mathrm{product}\;\left(g\right)}{\mathrm{carbon}\;\mathrm{in}\;\mathrm{initial}\;\mathrm{cellulose}\;\mathrm{sample}\;\left(g\right)}$$

(4) $$\mathrm{TOC}\;\mathrm{yield}\;\left(\%\right)=100\times \frac{\mathrm{carbon}\;\mathrm{in}\;\mathrm{aqueous}\;\mathrm{solution}\;\left(g\right)}{\mathrm{carbon}\;\mathrm{in}\;\mathrm{initial}\;\mathrm{cellulose}\;\mathrm{sample}\;\left(g\right)}$$

## 4. Conclusions

Ashless filter paper was dissolved in aqueous solutions of imidazolium ionic liquids containing alkyl side chains of 2 to 6 carbons at 373 K and aqueous bis(ethylenediamine ammonium) copper solution at room temperature, and was then precipitated by adding water as an anti-solvent. Then, the precipitated filter paper, pretreated by an IL, were hydrolyzed under hydrothermal conditions at 533 K and 5.0 MPa in a semi-batch reactor. The major products such as glucose, cello-oligosaccharides with DP = 2 to 9, fructose, 5-hydroxymethyl-2-furaldehyde, and levoglucosan were quantified. The hydrolytic dissolution rates in water were expressed by first order reaction kinetics. The rates treated with ILs were much higher than those without IL pretreatment except for [HMIM][Cl], while the crystallinity indexes were almost the same. For instance, the rate constant with [AMIM][Cl] at 533 K and 5.0 MPa was almost nine times as fast as that for untreated sample. The highest reaction rate and highest glucose and sugar yields were obtained with [AMIM][Cl], while final TOC yield was not the highest. While the rates with BEDC solution were higher than those with all ILs studied, the yields of glucose and cello-oligosaccharides were not the highest and the yields of further decomposition products increased. The yield of each product was proportional to the TOC yield, as seen for hydrolysis of pure cellulose without pretreatment.

The capital costs of hydrothermal reactors are costly, but the operating energy in terms of energy per mass of biomass is not so high because of the heat required for heating up liquid without the heat of vaporization (no phase change) and high reaction rate. Enzymes are also costly, depending on enzyme species. It is not easy to compare the energy consumption of both the methods. Process engineering analysis should be made for cost estimation for the comparison—the costs of construction, capital and operation for the process, as well as purification/separation of targeted compounds and ILs from the mixture of product solution. From a practical point of view, an IL should be recycled. The purification and recycling of ILs may need considerable energy. A further study is required for evaluating the processes for feasibility.

## Figures and Tables

**Table 1:** TOC and product yields from filter paper treated and untreated by various ILs.

**Pre-Treatment**						
**IL or BEDC**	**[EMIM][Cl]**	**[AMIM][Cl]**	**[BMIM][Cl]**	**[HMIM][Cl]**	**none**	**BEDC ^a^**
temperature (K)	373	373	373	373	-	298
time (min)	120	120	120	120	-	60
C.I. *X*_CI_	45	44	47	48	87	-
						
Hydrolysis						
temperature (K)	533	533	533	533	533	533
operation time (min)	60	60	60	60	60	60
TOC (%)	98.4	89.3	82.9	57.4	91.77	102.0
glucose	14.4	28.6	13.9	9.33	14.11	26.91
oligomer DP = 2 to 9	27.3	19.8	28.3	21.28	27.99	21.84
sugar ^b^	41.7	48.5	42.2	30.61	42.10	48.75
levoglucosan	1.19	3.16	1.34	0.93	2.01	24.0
5-HMF	3.67	4.88	2.67	1.58	5.35	5.85
fructose	2.50	1.89	2.05	1.36	3.00	3.47
DP = 2	8.84	6.49	10.40	7.89	16.56	10.76
3	6.30	5.97	7.59	4.72	4.63	5.89
4	4.19	3.28	4.19	3.26	3.03	2.54
5	3.06	1.67	2.52	2.21	1.52	1.22
6	1.98	1.02	1.46	1.28	1.01	0.73
7	1.52	0.71	1.10	0.98	0.55	0.41
8	0.85	0.33	0.69	0.59	0.41	0.18
9	0.58	0.37	0.39	0.35	0.29	0.10
Not identified product ^c^	49.3	30.9	34.7	23.0	39.3	1.6

^a^ Bis(ethylenediamine)copper(II) solution; ^b^ glucose + fructose + DP = 2 to 9; ^c^ difference between TOC yield and total yield of product measured.

**Table 2:** Chemicals employed with purities.

Compound	Purity (%)
1-Ethyl-3-methylimidazolium chloride ([EMIM][Cl])	98
1-Allyl-3-methylimidazolium chloride ([AMIM][Cl])	97.0
1-Butyl-3-methylimidazolium chloride ([BMIM][Cl])	98.0
1-Hexyl-3-methylimidazolium chloride ([HMIM][Cl])	97.0
d-(+)Glucose	99.5
d-(−)-Fructose	99.0
d-(+)-Cellobiose	98
d-(+)-Cellotriose	98
5-Hydroxylmethyl-2-furaldehyde	98
1,6-Anhydro-β-d-glucose	99
Bis(ethylenediamine)copper(II) solution (BEDC)	95
Cellulose filter paper	ashless, α-cellulose
